# Anti-Inflammatory and Antipruritic Effects of Remote Ischaemic Postconditioning in a Mouse Model of Experimental Allergic Contact Dermatitis

**DOI:** 10.3390/medicina59101816

**Published:** 2023-10-12

**Authors:** Ozgur Gunduz, Melike Sapmaz-Metin, Ruhan Deniz Topuz, Oktay Kaya, Cetin Hakan Karadag, Ahmet Ulugol

**Affiliations:** 1 Department of Medical Pharmacology, Faculty of Medicine, Trakya University, 22030 Edirne, Turkey; ruhantopuz@gmail.com (R.D.T.); hkaradag@trakya.edu.tr (C.H.K.); aulugol@trakya.edu.tr (A.U.); 2 Department of Histology and Embryology, Faculty of Medicine, Trakya University, 22030 Edirne, Turkey; melikesapmaz@yahoo.com; 3 Department of Physiology, Faculty of Medicine, Trakya University, 22030 Edirne, Turkey; oktaykaya@trakya.edu.tr

**Keywords:** allergic contact dermatitis, remote ischemic postconditioning, inflammation, pruritus, scratching behavior

## Abstract

*Background and Objectives:* Allergic contact dermatitis is a common type IV hypersensitivity reaction characterised by redness, itching, oedema and thickening of the skin. It occurs in about 7% of the population and its incidence is increasing. It has been observed that the preconditioning of tissues by exposing them to transient ischemia increases resistance to subsequent permanent ischemia, and this phenomenon is called ischemic preconditioning. It has been shown that conditioning in one organ can also protect other organs. The protective effect of remote ischemic preconditioning is thought to be based on the induction of anti-inflammatory responses. The aim of this project was to investigate the anti-inflammatory and antipruritic effects of remote ischemic postconditioning in a mouse model of experimental allergic contact dermatitis. *Methods:* Experimental allergic contact dermatitis was induced with 1-fluoro-2,4-dinitrobenzene. Remote ischemic postconditioning was performed at 3 and 25 h after the challenge. Ear thickness and number of scratches 24 and 48 h after challenge, as well as cytokine levels and the infiltration of mast cells, neutrophils, CD4^+^ and CD8^+^ T lymphocytes in serum and ear tissue at 48 h were measured to determine the effect of RIPsC. *Results:* Remote ischemic postconditioning decreased ear thickness, one of the symptoms of allergic contact dermatitis (*p* < 0.0001). It had no significant effect on the number of scratches. It reduced serum IL-17 levels (*p* < 0.01). It alleviated local inflammation by suppressing CD8^+^ T lymphocyte and neutrophil infiltration. *Conclusions:* It was concluded that remote ischemic postconditioning may alleviate the symptoms of allergic contact dermatitis by suppressing CD8^+^ T lymphocyte and neutrophil infiltration and reducing IL-17 secretion.

## 1. Introduction

Allergic contact dermatitis (ACD), considered a delayed hypersensitivity reaction, is a common skin disease [1]. It is estimated that 4 to 7% of dermatology consultations are for ACD. It is an important public health problem that negatively affects workplace productivity and socioeconomic status [2]. It occurs with signs and symptoms such as redness, itching, oedema, and oozing on the skin as a result of re-contact with the allergen [3]. T-cells, which are activated in response to the allergen during the sensitisation period, rapidly release their specific cytokines upon re-exposure to the allergen. Activated CD8^+^ T cells (Tc) generally have a pro-inflammatory effect, whereas CD4^+^ T cells (Th) can have a pro- or anti-inflammatory effect; that is, they have a regulatory function [4,5]. The effects of Tc and Th cell subtypes are the subject of research [6,7]. CD8^+^ Tc17 cells and CD4^+^ Th17 lymphocytes are involved in the elicitation phases [6]. Topical steroids, calcineurin inhibitors and systemic immunosuppressive agents are used in the treatment [8]. However, in some cases, existing treatments may be useless because they are ineffective and/or the frequency of adverse effects increases at their effective doses [9]. Therefore, effective new treatments are needed.

It is known that ischemic pre- or post-conditioning has a protective effect against ischemia/reperfusion injury [10,11]. İschemic conditioning of a remote organ has been shown to protect other organs affected by ischemia [12,13]. Remote ischemic preconditioning (RIPC) has been shown to have anti-inflammatory effects in rat models of acute inflammation, such as carrageenan-induced paw oedema, ifosfamide-induced haemorrhagic cystitis and indomethacin-induced gastric injury [14]. It was determined that RIPC caused anti-inflammatory effects by shifting the balance between CD4^+^ T lymphocytes Treg/T17 towards Treg in the necrotising enterocolitis mouse model [15]. It has been reported that remote ischemic postconditioning (RIPsC) plays a protective role by activating Treg in an ischemic stroke model, and that the protective effect of RIPsC is abolished in mice in which these cells are depleted [16].

The substances produced by ischemic conditioning and how they induce anti-inflammatory effects are being studied. Motomura et al. (2017) found that thioredoxin, macrophage migration inhibitory factor and dermcidin were increased in the sera of healthy volunteers undergoing RIPC [17]. Fukunaga et al. (2009) found that ear oedema, an indication of ACD-induced inflammation, was reduced in transgenic mice with a 10-fold increase in thioredoxin synthesis [18].

No studies have investigated the effects of ischemic conditioning on allergic contact dermatitis. We aimed to demonstrate the potential antipruritic and anti-inflammatory effects of RIPsC in female BALB/c mice by creating an experimental ACD model with 1-fluoro-2,4-dinitrobenzene (DNFB), counting scratching behaviour, measuring ear oedema, and determining cytokine levels and the involvement of mast, neutrophil, CD4^+^ and CD8^+^ T cells.

## 2. Materials and Methods

### 2.1. Animals and Ethics

This study was approved by Trakya University Animal Experiments Local Ethics Committee on 31 March 2021 with decision number 2021.03.04.

In this study, 32 female BALB/c mice with the same biological and physiological characteristics produced in Trakya University Experimental Animals Research Unit were used. They were housed in individually ventilated cages under standard laboratory conditions (22 ± 2 °C temperature, 12 h light/dark cycle and 45–65% relative humidity). Mice were given standard animal chow and tap water ad libitum.

### 2.2. Chemicals and Antibodies

1-fluoro-2,4-dinitrobenzene and olive oil from Sigma Aldrich (St. Louis, MO, USA), acetone from Merck (Darmstadt, Germany), anti-CD4 and anti-CD8 primary antibodies from Santa Cruz Biotechnology (Dallas, TX, USA), and ELISA kits from Nepenthe Research Technologies (Kocaeli, Turkey) were purchased.

### 2.3. Study Design

Four groups were established, including control, ACD, ACD + RIPsC (treatment) and ACD + ketamine/xylazine (K/X), and eight mice were randomised to each group. The experimental ACD model was established in both ears of mice using DNFB according to the method described by Ozlen et al. [19]. In the control group, only acetone/olive oil mixture (4:1) was applied.

After the challenge, RIPsC was administered to the treatment group at hour 3, when capillary vasodilation began, and at hour 25, when T lymphocyte infiltration increased. For the application of RIPsC, mice were anaesthetised with ketamine (100 mg/kg/i.p.)/xylazine (10 mg/kg/i.p.). After the adequate depth of anaesthesia, blood flow was stopped for 5 min by wrapping rubber bands around both hind legs (ischemia phase). The bands were then removed and the leg was reperfused for 5 min. Paleness of the hind paw during rubber band occlusion and reactive hyperemia during reperfusion were observed. The ischemia–reperfusion cycle was performed three times [20,21]. Animals in the ACD + K/X group (anaesthesia group) were anaesthetised at 3 and 25 h, but RIPsC was not administered.

**Figure 1:**
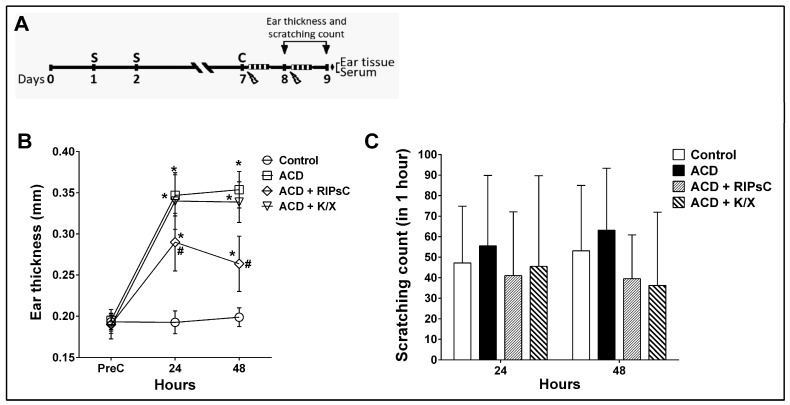
Study design (**A**); effect of remote ischemic postconditioning on ear thickness (**B**), and scratching count (**C**). S: sensitisation; C: challenge; 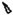: anaesthesia procedure; 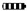: ischemic postconditioning (5 min ischemia, 5 min reperfusion/3 cycles); ♦: euthanasia; ACD: allergic contact dermatitis; RIPsC: remote ischemic postconditioning; K/X: ketamine/xylazine. * *p* < 0.0001 compared with control group; # *p* < 0.0001 compared with ACD group. Two-way analysis of variance, post hoc Bonferroni test. Data are expressed as mean ± SD (n = 16 ears, n = 8 mice).

In all groups, ear thickness and number of scratches were assessed at 24 and 48 h after challenge. Following the 48 h measurements, euthanasia was performed by taking blood from the heart under anaesthesia. The study design is shown in Figure 1A. Blood was collected in a dry tube for 30 min and centrifuged at 2000 rpm for 20 min. The serum samples obtained were dispensed into 1 mL microtubes for ELISA measurements and stored at −80 °C. Both auricles were rapidly removed. The right auricles were weighed, placed in microtubes and stored at −80 °C until ELISA measurements; the left auricles were fixed in neutral formaldehyde and separated for immunohistochemistry and histology.

### 2.4. Measurement of Scratching Behaviour

The scratching behaviour of the mice at 24 and 48 h after the challenge was recorded with a video camera for 1 h in a sound-insulated room. Mice exhibit several scratching behaviours per second. This behaviour was here considered a bout of itching. Scratching bouts were then counted from the recording by the same investigator, and compared with the control group [19,22].

### 2.5. Measurement of Ear Thickness

Ear thickness was measured by the same investigator using a digital caliper (Accud, Istanbul, Turkey) with a resolution of 0.01 mm and an accuracy of ±0.04 mm before and 24 and 48 h after the challenge [19,23].

### 2.6. Measurement of Cytokine Levels

Ear tissues were homogenised according to the method described by Ozlen et al. [19]. Samples were centrifuged at 14,000 rpm for 20 min at 4 °C, and the supernatants were separated for protein and cytokine measurements. Protein concentrations were measured in mg/mL using a nanodrop device (Mecasys, Daejeon, Republic of Korea). Interferon-γ (IFN-γ), interleukin 2 (IL-2) and IL-17 cytokine concentrations were measured using a sandwich ELISA kit according to the manufacturer’s recommendations (Nepenthe, Kocaeli, Turkey). Tissue cytokine concentrations were expressed as pg or ng/mg protein, and serum cytokine concentrations were expressed as pg or ng/mL. The detection limits of the IFN gamma, IL-4, and IL-7 ELISA kits are 10–640 pg/mL, 12.5–800 pg/mL, and 10–640 pg/mL, respectively.

### 2.7. Histological Analysis

The upper parts of the left auricles of the mice were fixed in 10% neutral formalin for 24 h. Sections of 5 μm thickness were cut from the resulting paraffin blocks. Haematoxylin and eosin (H&E) staining was used to show the histological structure of the ear, and toluidine blue staining was used to assess the number of mast cells (MC).

H&E-stained sections were analysed using a light microscope (Olympus BX51) at 40×–100×–200×–400× magnification to compare the histological findings of the ear in all groups. A histological damage score was calculated based on hyperkeratosis, apoptotic changes in keratinocytes, lymphocyte/leukocyte exocytosis, spongiosis in the epidermis, lymphocytic and neutrophilic infiltration, follicular infiltration, vasodilatation and perivascular infiltration in the dermis caused by ACD (0: no change; 1: mild; 2: moderate; 3: severe; and 4: very severe) [24].

The total thickness of the epidermis and dermis was measured using an Olympus BX51 light microscope at 200× magnification on H&E-stained sections using AxioVision Rel 4.8 software by two blinded observers.

The distribution of mast cells was counted in the subepidermal and perifollicular dermis at a magnification of 400× in 10 randomly selected microscopic fields per section for each subject using an Olympus BX51 microscope (Tokyo, Japan). The results are expressed as total cell counts [25].

### 2.8. Immunohistochemical Analysis

Paraffin sections of 5 μm obtained from control and ACD subjects were deparaffinised after overnight incubation at 56 °C. Sections were rehydrated through a series of alcohol solutions. For antigen retrieval, citrate buffer (pH 6.0, Thermo Scientific, Waltham, MA, USA) was boiled for 10 min. Endogenous peroxidase activity was blocked by incubation with 3% hydrogen peroxide (H_2_O_2_) for 10 min. Sections were treated with 1% pre-immune rabbit serum (Thermo Scientific, Ultra V Block, TA-125-UB, Waltham, MA, USA) for 10 min to block non-specific antibody binding. Ear sections were incubated with anti-CD4 (Santa Cruz, MT310, sc-19641, 1:50 dilution) and anti-CD8 (Santa Cruz, D-9, sc-7970, 1:50 dilution) primary antibodies overnight at +4 °C. After washing with phosphate buffer (PBS), secondary antibodies (Thermo Scientific, Biotinylated Goat anti-Polyvalent, TP-125-BN, Waltham, MA, USA) were applied for 10 min at room temperature. The sections were exposed to HRP-streptavidin (Thermo Scientific, Streptavidin peroxidase, TS-125-HR, Waltham, MA, USA) for 10 min and, after washing with PBS, DAB chromogen was applied. Tissues stained with Mayer’s hematoxylin in the post-field were passed through alcohol series and coverslipped with Entellan. CD4^+^- and CD8^+^-positive cells were counted in 10 randomly selected areas in the sections from each subject at 400× magnification using an Olympus BX51 microscope (Tokyo, Japan). The results are expressed as total cell counts.

### 2.9. Statistical Analysis

GraphPad Prism version 6.0c (GraphPad Software Inc., La Jolla, CA, USA) was used for statistical analysis. Two-way analysis of variance and post hoc Bonferroni test were used to evaluate the changes in ear thickness and itching counts according to time. Epidermis + dermis thickness, tissue and serum cytokine levels, CD4^+^ and CD8^+^ T-cell counts, mast cell counts and tissue damage score were evaluated by one-way analysis of variance followed by post hoc Bonferroni test. Data are expressed as mean ± standard deviation (SD). *p* < 0.05 was considered significant.

## 3. Results

### 3.1. Effect of Remote Ischemic Postconditioning on Ear Thickness and Itching Behaviour

At 24 and 48 h post-challenge, ear thickness increased in the ACD group compared to the control group (Figure 1B, * *p* < 0.0001). RIPsC significantly reduced ear thickness compared to the ACD group at both 24 h and 48 h (Figure 1B, # *p* < 0.0001). In the ACD group, the number of scratches was not statistically significant (Figure 1C). RIPsC had no effect on the number of scratches (Figure 1B).

### 3.2. Effect of Remote Ischemic Postconditioning on Tissue and Serum Cytokine Concentrations

**Figure 2:**
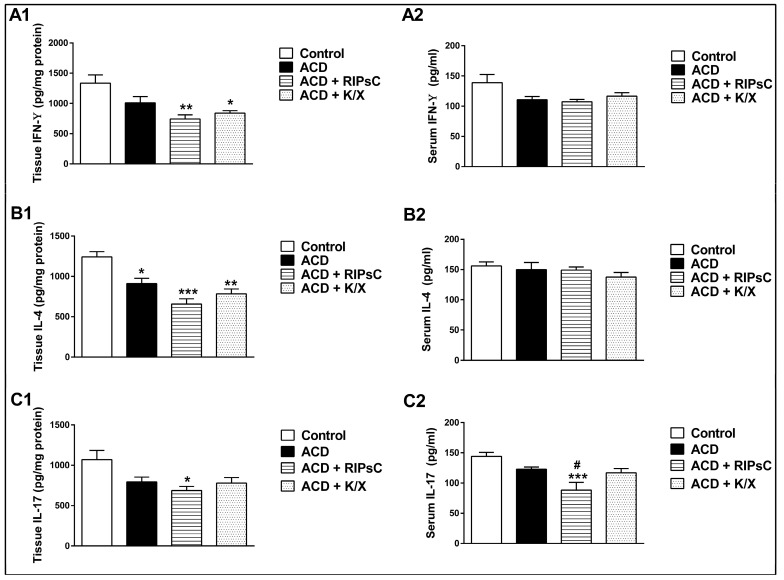
Effect of remote ischemic postconditioning on tissue (**A1**–**C1**) and serum (**A2**–**C2**) cytokine levels. PSNL; ACD: allergic contact dermatitis; RIPsC: remote ischemic postconditioning; K/X: ketamine/xylazine. * *p* < 0.01; ** *p* < 0.001, *** *p* < 0.0001 compared with control group; # *p* < 0.05 compared with ACD group. One-way analysis of variance, post hoc Bonferroni test. Data are expressed as mean ± SD (n = 8).

Ear tissue IFN-γ levels were significantly lower in the ACD + RIPsC and ACD + K/X groups compared to the control group at 48 h post-challenge (Figure 2A1, ** *p* < 0.001 and * *p* < 0.01, respectively). There were no significant differences in serum IFN-γ levels between groups at 48 h post-challenge (Figure 2A2).

Ear tissue IL-4 levels were significantly lower in the ACD, ACD + RIPsC and ACD + K/X groups compared to the control group at 48 h (Figure 2B1, * *p* < 0.01, *** *p* < 0.0001 and ** *p* < 0.001, respectively). No differences in serum IL-4 levels were observed between groups at 48 h post-challenge (Figure 2B2).

At 48 h, ear tissue IL-17 levels were significantly lower in the ACD + RIPsC group than in the control group (Figure 2C1, * *p* < 0.01). At 48 h post-challenge, serum IL-17 levels were significantly lower in the ACD + RIPsC group compared to the control group (Figure 2C2, *** *p* < 0.001). RIPsC decreased the serum IL-17 levels compared to the ACD group (Figure 2C2, # *p* < 0.05).

### 3.3. Effect of Remote Ischemic Postconditioning on Histological Changes

#### 3.3.1. Haematoxylin & Eosin Staining

**Figure 3:**
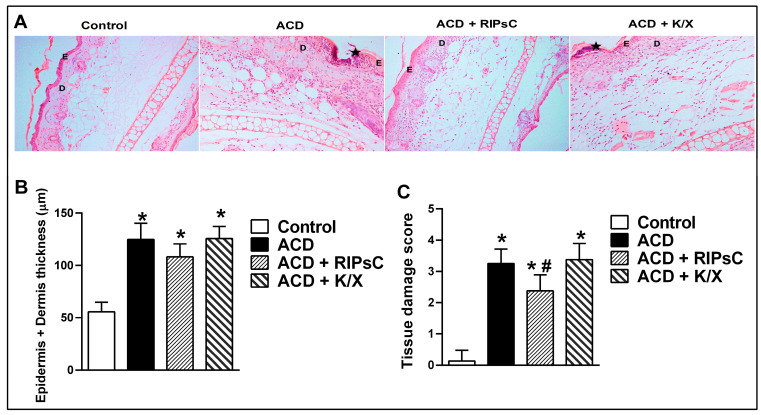
Micrographs of ear tissue from control and all ACD groups, Haematoxylin–Eosin, 200× (**A**), epidermis + dermis total thickness (**B**) and tissue damage score (**C**). ACD: allergic contact dermatitis; RIPsC: remote ischemic postconditioning; K/X: ketamine/xylazine. E: epidermis, D: dermis. Asterisk: exocytosis focus. * *p* < 0.0001 compared to the control group; # *p* < 0.01 compared to the ACD group one-way analysis of variance, post hoc Bonferroni test. Data are presented as mean ± SD (n = 7–8).

H&E-stained ear tissue sections from control group mice showed normal epidermal and dermal layers, muscle and cartilage. There was a thin layer of keratin overlying the epidermis. In the epidermis of the ACD group, spongiosis was seen between the basal keratinocytes; pyknotic nuclei and leukocyte exocytosis mainly containing neutrophils and hyperkeratosis were observed. Vascular dilation, interstitial oedema and neutrophilic infiltration were prominent in the dermis. In the ACD + RIPsC group, exocytosis in the epidermis, keratinocyte apoptosis, vasodilatation and infiltration in the dermis were alleviated. Sections from the ACD + K/X group showed similar findings to the ACD group (Figure 3A).

The results of the epidermis and dermis thickness measurements of the groups are shown in Figure 3B. In all ACD groups (ACD, ACD + RIPsC and ACD + K/X), epidermis and dermis thickness was significantly higher than in the control group (Figure 3B, * *p* < 0.0001).

Tissue damage caused by ACD on H&E sections was scored according to histopathological findings, and the results are shown in Figure 3C. In all ACD groups (ACD, ACD + RIPsC and ACD + K/X), the tissue damage score was significantly higher than in the control group (Figure 3C, * *p* < 0.0001).

RIPsC improved ACD-induced tissue damage in the ear tissue by reducing interstitial oedema and apoptotic cell death (Figure 3C, # *p* < 0.01).

#### 3.3.2. Toluidine Staining

Figure S1 shows mast cells labeled with toluidine blue. Mast cells showing metachromasia were observed in the dermis and perifollicular areas (Figure S1A). No significant difference was found between the groups when the number of mast cells was evaluated (Figure S1B).

### 3.4. Effect of Remote Ischemic Postconditioning on T-Cell Infiltration

**Figure 4:**
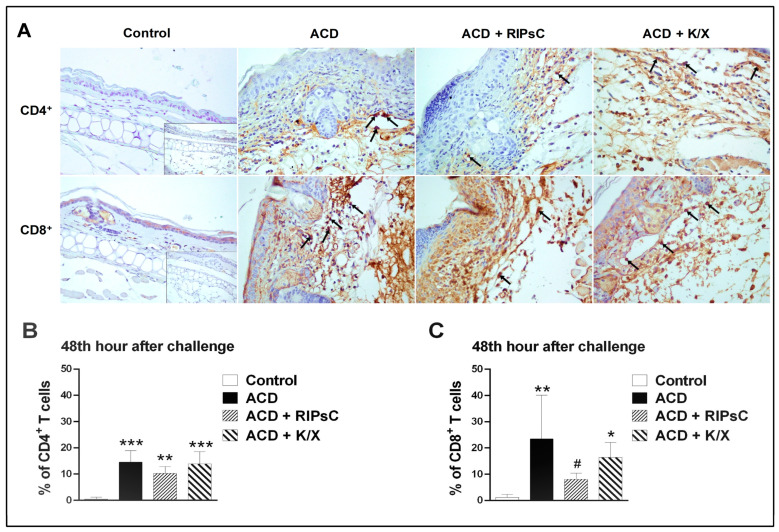
Immunohistochemical staining results for CD4^+^ and CD8^+^ T lymphocytes in ear tissue. CD4^+^ and CD8^+^ immunopositive cells are observed in the dermis in all ACD groups. Inset: negative controls. Haematoxylin counterstaining, 400× (**A**), CD4^+^ T-cell counts (**B**) and CD8^+^ T-cell counts (**C**). ACD: allergic contact dermatitis; RIPsC: remote ischemic postconditioning; K/X: ketamine/xylazine. Arrow: positively stained CD4^+^/CD8^+^ T lymphocytes. * *p* < 0.05, ** *p* < 0.001, *** *p* < 0.0001 compared with control group; # *p* < 0.05 compared with ACD group. One-way analysis of variance, post hoc Bonferroni test. Data are expressed as mean ± SD (n = 7).

CD4^+^ and CD8^+^ immunostaining was performed to determine the role of lymphocytes in the ACD animal model (Figure 4). In the prepared sections, cells stained brown cytoplasmically with DAB were considered immunopositive for CD4^+^ and CD8^+^. No positively stained CD4^+^ and CD8^+^ T cells were observed in the epidermis of the control group and all ACD groups. CD4^+^ T cells were found in perivascular and intravascular areas in the dermis (Figure 4A). CD8^+^ T cells were observed perivascularly in the dermis (Figure 4A).

The CD4+ T cell counts of the groups are shown in Figure 4B and the CD8^+^ T cell counts are shown in Figure 4C. In all ACD groups (ACD, ACD + RIPsC, ACD + K/X), CD4^+^ and CD8^+^ cell counts in the dermal layer were significantly higher than in the control group (* *p* < 0.05, ** *p* < 0.001, *** *p* < 0.0001, respectively). Remote ischemic postconditioning decreased CD8^+^ T cell infiltration (Figure 4C, # *p* < 0.05).

## 4. Discussion

ACD is a common skin condition that affects quality of life. Despite its prevalence, its pathogenesis is not fully understood. The most effective treatment is to avoid the identified allergen, but this is not always possible. It is not possible to determine when and under what conditions sensitisation to an allergen occurs. Symptoms such as skin erythema, oozing, vesicles and itching begin to appear on the third day after re-exposure to the antigen (challenge). In experimental animal models of ACD, this period is shorter, with post-challenge symptoms beginning at 3 h and peaking at 48 h [26].

ACD treatment is usually initiated after the onset of symptoms. Therefore, in our study, the first administration of RIPsC was given three hours after the challenge when the first symptom (vasodilation) was observed. The second administration of RIPsC was given at hour 25 when lymphocyte infiltration peaked [27]. In this way, an application similar to routine treatment was achieved.

In our study, the ACD model was induced in BALB/c female mice with DNFB. After the challenge, a significant increase in ear thickness, an indicator of oedema, was observed in the ACD group at 24 and 48 h. Saika et al. found an average increase in ear thickness of 150 µm in the ACD group in their study using DNFB in female BALB/c mice [28]. Liu et al. found an increase in ear thickness of approximately 200 µm in the ACD model induced by DNFB in mice [7]. Our study found an increase in ear thickness of approximately 150 µm in the ACD group compared to baseline measurements. Our ear thickness measurements are similar to those in previous studies.

We found that epidermis–dermis thickness and histological damage score were higher in the ACD group compared to the control group. We determined the total thickness of the epidermis and dermis in the ACD group to be 130 µm on average. In their study, Zaladonis et al. also found an increase in epidermal plus dermal thickness in the ACD group, but measured the total thickness as 150–200 µm. This difference may be due to the use of C57BL/6J mice in their study [29].

We found that RIPsC prevented the development of ear oedema at both 24 and 48 h. In the ACD + RIPsC group, vasodilatation in the dermis and neutrophil infiltration were reduced in ear tissue collected at 48 h. We also found that RIPsC reduced CD8^+^ T lymphocyte infiltration but did not affect mast cell numbers. RIPsC may have alleviated local inflammation by reducing neutrophil and CD8^+^ T lymphocyte infiltration. Reduced inflammation, capillary vasodilatation and permeability may attenuate oedema formation [30]. In our study, mast cells were not found to play a role in the capillary vasodilatation that developed in the first 48 h.

Scratching behaviour was similar between the groups. The fact that the increasing trend in the number of scratches in the ACD group did not reach statistical significance was thought to be related to the inadequate number of subjects or the use of a single challenge. To determine the effects of RIPsC on the number of scratches, further studies on the ACD model with repeated challenges are required. It was concluded that the lack of baseline scratch counts in our study was a significant limitation.

Contrary to expectations, both tissue and serum levels of IFN-γ, IL-4 and IL-17 did not increase in the ACD group. The effect of RIPsC on cytokine levels showed a decrease in all cytokines except serum IFN-γ and IL-4 levels compared to the control group. RIPsC was found to decrease serum IL-17 levels compared to the ACD group. He et al. showed that the neutralisation of IL-17 was more effective than the neutralisation of IFN-γ in preventing oedema [6]. IL-17 is classified as a pro-inflammatory cytokine involved in the pathogenesis of ACD [31]. CD8^+^ TIL-17 cells are the main cells that synthesise IL-17. RIPsC may act through CD8^+^ TIL-17 cells.

Balaha et al. calculated the histological damage score using a 0–3 scale for epidermal hyperplasia, hyperkeratosis, occluded blood vessels and inflammatory cell infiltration parameters in H&E-stained sections in the ACD model induced by DNFB in mice. The researchers found that the damage score was higher in the ACD group compared to the control group, similar to our study’s data [32]. In our study, we found that the application of RIPsC alleviated the histological damage caused by ACD.

Immunostaining showed CD4^+^ T cells in the perivascular and intravascular areas of the dermis and CD8^+^ T cells in the perivascular areas of the dermis. CD4^+^ and CD8^+^ lymphocyte infiltration was shown to be increased in the ACD group. During the symptomatic phase of ACD, re-exposure to hapten results in keratinocyte apoptosis, inflammation and dermal oedema mediated by effector CD8^+^ T cells [33]. The administration of RIPsC may attenuate ACD-induced inflammation and apoptotic changes by suppressing CD8^+^ T cell infiltration.

Glucocorticoids are potent anti-inflammatory drugs commonly used in the treatment of inflammatory skin diseases [9]. They are highly effective on ear edema and epidermis thickness, which are findings yielded by ACD [34]. Adverse effects such as muscle or skin atrophy, striae, rubeosis or acne limit their use [9]. Alternative therapies are being investigated [35,36]. To the best of our knowledge, our study is the first investigating the effects of RIPsC in an experimental ACD model.

There are randomised–controlled clinical studies showing that RIPsC is effective in the treatment of diseases such as ischemic stroke and subcortical ischemic vascular dementia [37,38]. We think that if the positive effects of RIPsC on ACD are supported by randomised–controlled clinical trials, it may be considered as a treatment alternative.

One of the important limitations of our study is that the efficacy and adverse effects of RIPsC were not compared with steroids. Other major limitations of our study are that baseline scratching levels were not counted, and chronic ACD model groups with repeated challenge were not included.

## 5. Conclusions

In conclusion, our study is the first to investigate the effects of RIPsC on allergic contact dermatitis, and we found that RIPsC alleviated local inflammation and prevented ear oedema by reducing neutrophil and CD8^+^ T lymphocyte infiltration and serum IL-17 levels. We also found that RIPsC reduced epidermis–dermis thickness and the histological damage score. Further studies to elucidate the mechanism by which RIPsC prevents CD8^+^ T cell infiltration and to determine CD4^+^ and CD8^+^ T lymphocyte subtypes will contribute to the understanding of the course of ACD.

## Data Availability

The data presented in this study are available on request from the corresponding author. The data are not publicly available due to privacy.

## Supplementary Materials

The following supporting information can be downloaded at: https://www.mdpi.com/article/10.3390/medicina59101816/s1, Figure S1. Micrographs showing mast cell distribution in the ear tissue with toluidine blue staining.
