# Supplementary material for: Anti-Inflammatory and Antipruritic Effects of Remote Ischaemic Postconditioning in a Mouse Model of Experimental Allergic Contact Dermatitis
Source: Medicina (Kaunas). 2023 Oct 12;59(10):1816. doi: 10.3390/medicina59101816 (PMC10608757; doi:10.3390/medicina59101816)
Supplement: Supplementary file 1 [file medicina-59-01816-s001.zip › medicina-2609374-supplementary.pdf]

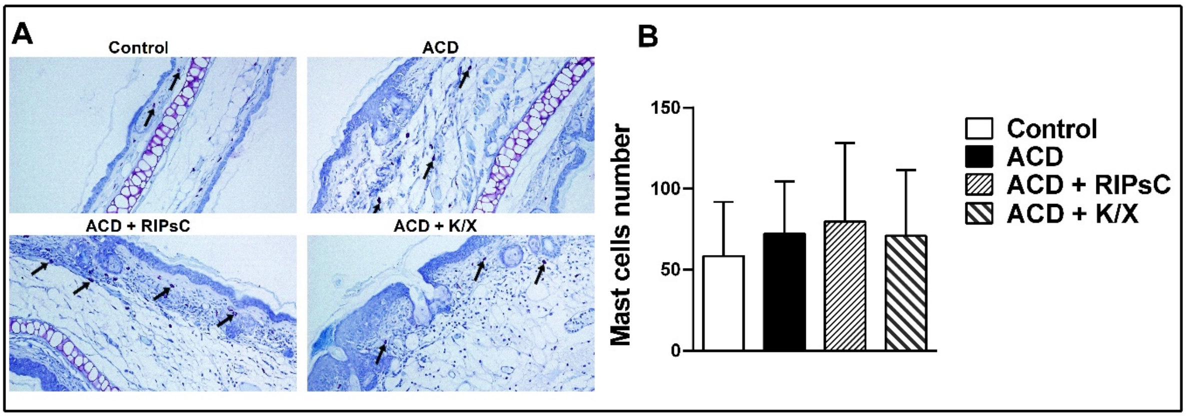

Figure S1. Micrographs showing mast cell distribution in the ear tissue with toluidine blue staining, 400× (**A**) and mast cell numbers (**B**). ACD: allergic contact dermatitis; RIPsC: remote ischemic postconditioning; K/X: ketamine/xylazine. Arrow: mast cell. One-way analysis of variance, post hoc Bonferroni test. Data are expressed as mean ± SD (n = 8).
